# Supplementary material for: CUT&Tag recovers up to half of ENCODE ChIP-seq histone acetylation peaks
Source: Nat Commun. 2025 Mar 27;16:2993. doi: 10.1038/s41467-025-58137-2 (PMC11950320; doi:10.1038/s41467-025-58137-2)
Supplement: Supplementary file 1 — Supplementary Information [file 41467_2025_58137_MOESM1_ESM.pdf]

Supplementary Materials

Supplementary Tables

Gene	Control type	Primer sequence (5’→3’)
ARHGAP22	Positive	Fw: GCTGAGAAGGAAGGGCTTAAT<br>Rv: GCTAGTCGGGATGATTTACAGG
COX4I2	Positive	Fw: GGATACCTCCAAGGCTTCATAC<br>Rv: GTAGTCACAGAACTAGGGTTGG
MTHFR	Positive	Fw: GGGTGGAACATCTCGAACTATC<br>Rv: GAACGAAGCCAGAGGAAACA
ZMYND8	Positive	Fw: GGATCTACAAACTTCCCTTCCC<br>Rv: GAAGGCATCGCAGGCTAATA
KLHL11	Negative	Fw: GACAAGCAGTGGCTCTACAA<br>Rv: CAGTATCGGAAAGAAGCCTACC
SIGIRR	Negative	Fw: CCAAGCTCAGACCTCAAAGT<br>Rv: TTCTTGCTGTGCTCGTATCC

Supplementary Table 1. qPCR primer sequences. Control primer sequences based on ENCODE peaks.

Fw: forward; rv: reverse.

Sample	Total Fragments	Mapped Fragments	Alignment Rate	Duplication Rate	Unique Fragments	% chrM Fragments
Active Motif 39133 1:100 15 PCR SDS	34,467,445	34,246,137	99.36%	98.45%	531,603	9.14
Diagenode C15410196 1:100 15 PCR SDS	13,970,902	13,884,243	99.38%	89.15%	1,506,260	11.71
Diagenode C15410196 1:50 15 PCR SDS	90,812,784	83,771,834	92.25%	96.36%	3,051,389	3.16
Abcam-ab177178 1:100 15 PCR SDS	7,911,303	7,839,891	99.10%	55.49%	3,489,316	0.34
Abcam-ab4729 1:100 15 PCR SDS	146,621,482	134,760,046	91.91%	96.94%	4,123,523	1.03
CST9733 1:100 15 PCR SDS	9,912,113	9,823,687	99.11%	57.12%	4,211,994	0.25
Active Motif 39133 1:100 11 PCR SDS	4,380,560	4,347,855	99.25%	81.68%	796,514	6.39
Diagenode C15410196 1:50 11 PCR SDS	6,379,299	6,315,402	99.00%	85.67%	904,726	8.45
Abcam-ab177178 1:100 11 PCR SDS	21,349,457	21,233,210	99.46%	82.97%	3,615,527	0.59
Abcam-ab4729 1:100 11 PCR SDS	9,195,139	9,136,507	99.36%	80.53%	1,779,088	2.58
H3K27me3 11 PCR SDS	17,976,126	17,862,415	99.37%	88.75%	2,009,039	1.50
Active Motif 39133 1:100 11 PCR column	1,475,470	1,464,695	99.27%	46.33%	786,081	7.70
Diagenode C15410196 1:50 11 PCR column	1,800,855	1,785,868	99.17%	52.74%	843,989	12.40
Abcam-ab177178 1:100 11 PCR column	2,733,847	2,713,351	99.25%	51.59%	1,313,493	1.70
Abcam-ab4729 1:100 11 PCR column	2,153,420	2,140,283	99.39%	51.21%	1,044,240	2.73
H3K27me3 11 PCR column	3,594,729	3,563,911	99.14%	48.88%	1,822,039	1.64
Abcam-ab177178 1:100 13 PCR SDS	8,521,987	8,436,844	99.00%	73.70%	2,219,025	0.66
Abcam-ab4729 1:100 13 PCR SDS	10,601,213	10,513,132	99.17%	85.88%	1,484,538	2.29
Active Motif 39133 1:100 13 PCR SDS	9,424,194	9,368,250	99.41%	83.89%	1,508,766	12.90
CST9733 1:100 13 PCR SDS	25,021,566	24,909,845	99.55%	84.24%	3,926,215	0.54
Diagenode C15410196 1:50 13 PCR SDS	9,497,265	9,431,247	99.30%	79.54%	1,929,369	9.76
Abcam-ab177178 1:100 13 PCR column	10,658,973	10,607,882	99.52%	77.48%	2,389,150	0.55
Abcam-ab4729 1:100 13 PCR column	4,908,403	4,878,543	99.39%	81.33%	910,834	2.10
Active Motif 39133 1:100 13 PCR column	5,617,892	5,575,622	99.25%	87.05%	722,071	12.54
CST9733 1:100 13 PCR column	14,268,256	14,197,536	99.50%	82.79%	2,442,942	0.98
Diagenode C15410196 1:50 13 PCR column	5,168,457	5,110,735	98.88%	85.17%	758,019	16.85
Diagenode C15410196 1:100 15 PCR SDS TSA	3,157,488	3,133,254	99.23%	75.22%	776,367	1.65
Diagenode C15410196 1:50 15 PCR SDS TSA	3,922,942	3,879,429	98.89%	70.88%	1,129,659	8.98
Abcam-ab177178 1:100 15 PCR SDS TSA	5,178,780	5,132,446	99.11%	77.70%	1,144,541	8.61
Abcam-ab4729 1:100 15 PCR SDS TSA	12,570,714	12,473,438	99.23%	64.56%	4,420,973	0.71

Supplementary Table 2. Sequencing and alignment metrics of CUT&Tag data in experimental optimization.

SDS: sodium dodecyl sulfate; TSA: trichostatin A.

Sample	Total Fragments	Mapped Fragments	Alignment Rate	Duplication Rate	Unique Fragments	% chrM Fragments
Abcam-ab177178-1 1:100 15 PCR SDS	8,749,603	8,599,574	98.29%	13.94%	7,400,475	1.74
Abcam-ab177178-2 1:100 15 PCR SDS	8,754,917	8,585,036	98.06%	14.22%	7,364,313	1.68
Abcam-ab4729-1 1:100 15 PCR SDS	6,420,162	6,287,558	97.93%	30.04%	4,398,945	1.90
Abcam-ab4729-2 1:100 15 PCR SDS	5,963,254	5,797,807	97.23%	17.99%	4,754,984	1.53
Diagenode-1 C15410196 1:50 15 PCR SDS	6,625,752	6,410,793	96.76%	33.07%	4,290,718	10.52
Diagenode-2 C15410196 1:50 15 PCR SDS	9,790,708	9,528,668	97.32%	32.32%	6,448,536	9.42
CST 9733-1 1:100 15 PCR SDS	7,382,869	7,272,406	98.50%	11.49%	6,436,828	0.81
CST 9733-2 1:100 15 PCR SDS	9,392,608	9,254,221	98.53%	12.41%	8,105,633	0.68
H3K27ac Kaya-Okur-1 SRR8383507 (C&T)	2,471,858	2,293,087	92.77%	20.18%	1,830,390	2.02
H3K27ac Kaya-Okur-2 SRR8383508 (C&T)	3,320,561	3,088,577	93.01%	14.87%	2,629,278	2.00
H3K27ac Meers SRR8581604 (C&R)	6,777,196	5,523,031	81.49%	7.30%	5,120,026	0.10
H3K27me3 Kaya-Okur-1 SRR11074238 (C&T)	3,945,633	3,806,740	96.48%	1.43%	3,752,159	0.06
H3K27me3 Kaya-Okur-2 SRR11074239 (C&T)	4,159,984	4,051,439	97.39%	1.36%	3,996,319	0.04
H3K27me3 Meers SRR9073702 (C&R)	9,047,596	8,668,833	95.81%	1.58%	8,531,865	0.02

Supplementary Table 3. Sequencing and alignment metrics of CUT&Tag data used for ENCODE ChIP benchmarking.

C&R: CUT&RUN; C&T: CUT&Tag; SDS: sodium dodecyl sulfate.

Supplementary Figures

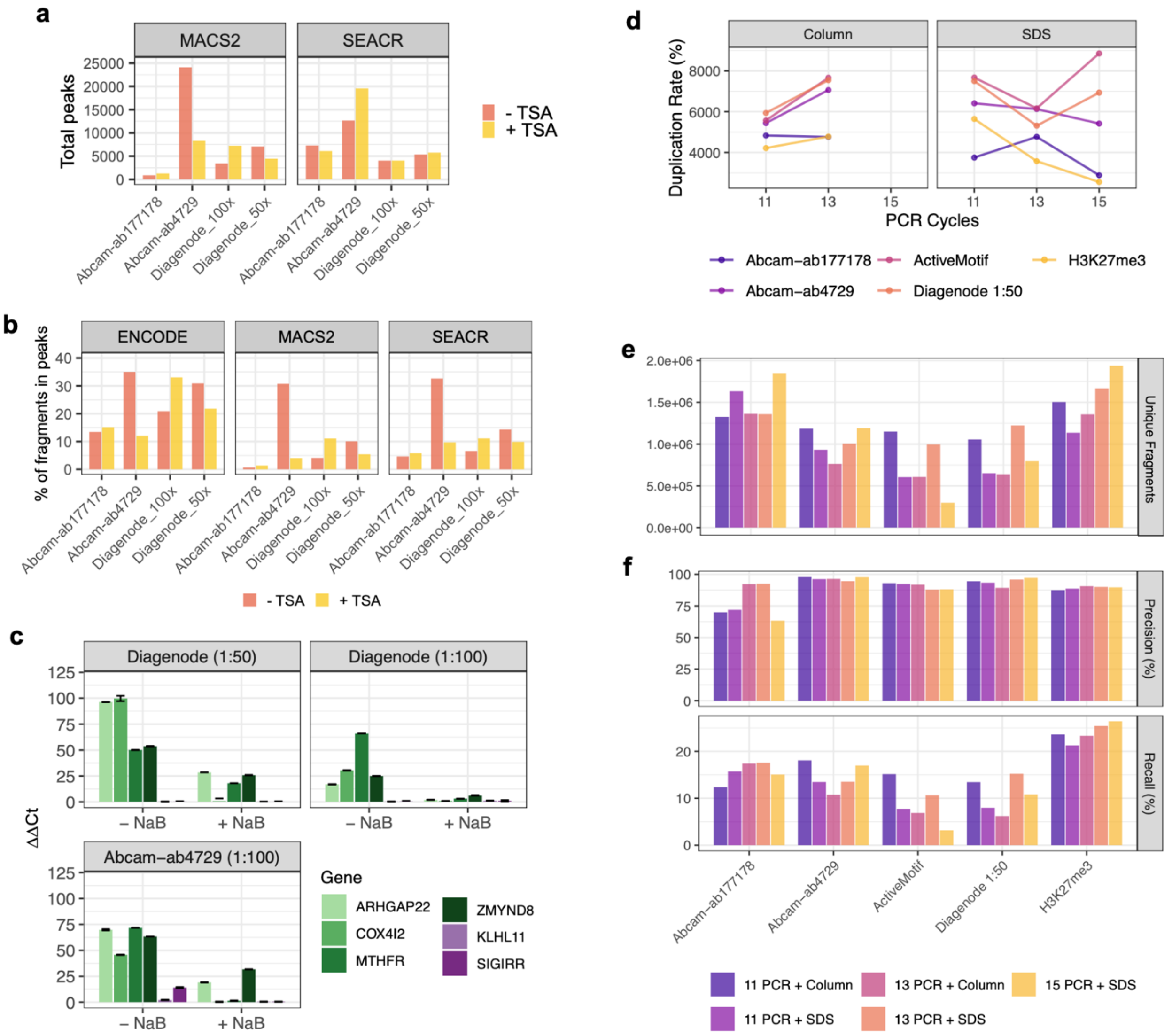

Supplementary Figure 1. Experimental optimization of CUT&Tag. a-b) Total peaks called (**a**) and FRiP scores (**b**) obtained in both called sample peaks, called with MACS2 and SEACR, and ENCODE H3K27ac peaks with and without treatment with TSA. **c)** Results of qPCR amplification of genes in most significant ENCODE H3K27ac peak regions (positive controls; green) versus least significant (negative controls; purple) in CUT&Tag experiments performed with top-performing antibodies, with and without HDAC inhibitor sodium butyrate (NaB; 5 mM). **d-f)** Duplication rates (**d**), total unique fragments (**e**), and ENCODE capture (**f**) metrics obtained using column- or SDS-

based DNA extraction and 11, 13 or 15 PCR cycles for sequencing library preparation, with all samples down sampled to the same read depth of 2.6 million paired-end reads with SEACR peak calling.

SDS: sodium dodecyl sulfate; TSA: trichostatin A.

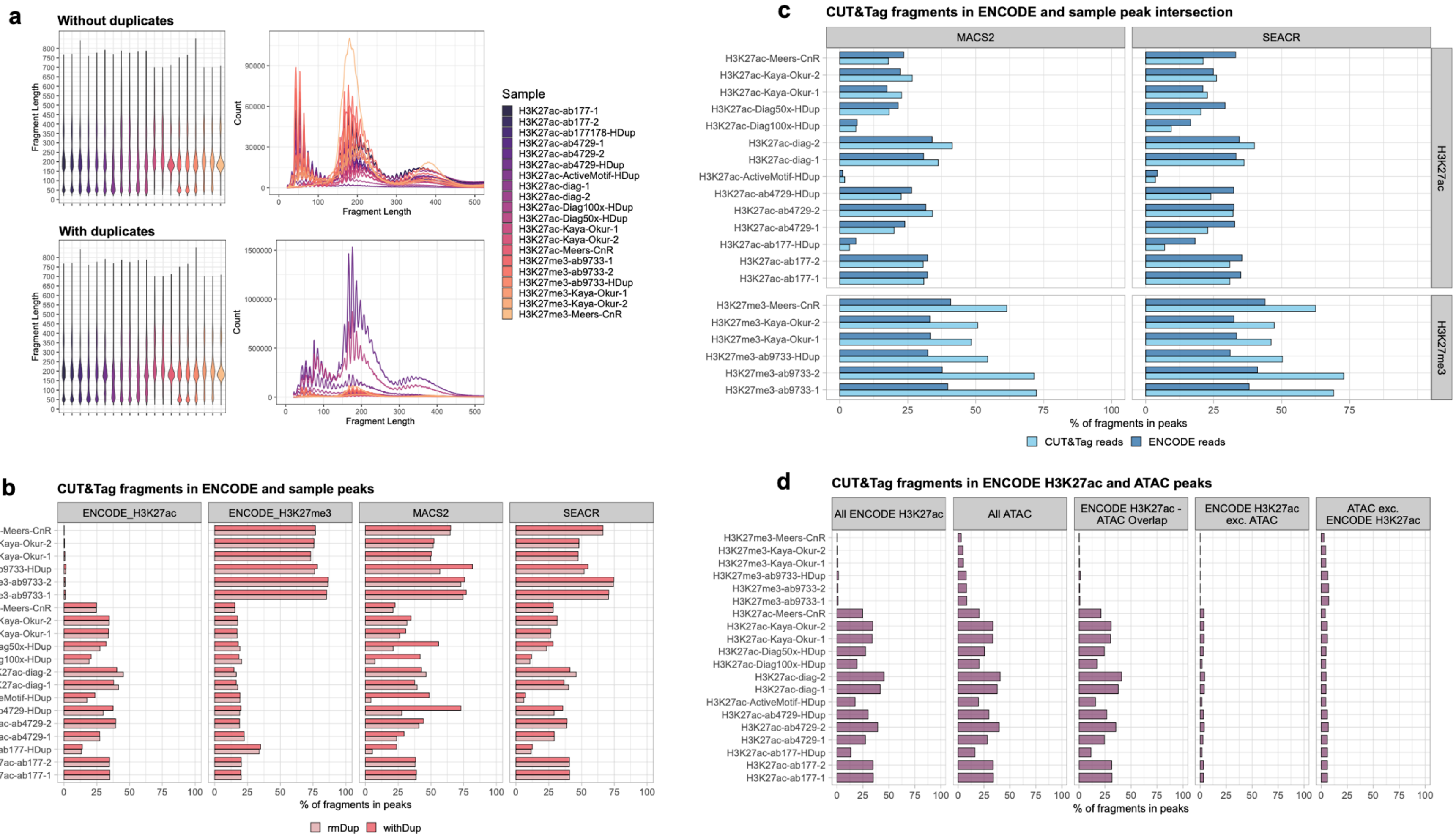
a
Without duplicates
With duplicates
Fragment Length
Count
Sample
H3K27ac-ab177-1
H3K27ac-ab177-2
H3K27ac-ab177178-HDup
H3K27ac-ab4729-1
H3K27ac-ab4729-2
H3K27ac-ab4729-HDup
H3K27ac-ActiveMotif-HDup
H3K27ac-diag-1
H3K27ac-diag-2
H3K27ac-Diag100x-HDup
H3K27ac-Diag50x-HDup
H3K27ac-Kaya-Okur-1
H3K27ac-Kaya-Okur-2
H3K27ac-Meers-CnR
H3K27me3-ab9733-1
H3K27me3-ab9733-2
H3K27me3-ab9733-HDup
H3K27me3-Kaya-Okur-1
H3K27me3-Kaya-Okur-2
H3K27me3-Meers-CnR
b
CUT&Tag fragments in ENCODE and sample peaks
ENCODE_H3K27ac
ENCODE_H3K27me3
MACS2
SEACR
% of fragments in peaks
rmDup
withDup
c
CUT&Tag fragments in ENCODE and sample peak intersection
MACS2
SEACR
H3K27ac
H3K27me3
% of fragments in peaks
CUT&Tag reads
ENCODE reads
d
CUT&Tag fragments in ENCODE H3K27ac and ATAC peaks
All ENCODE H3K27ac
All ATAC
ENCODE H3K27ac - ATAC Overlap
ENCODE H3K27ac exc. ATAC
ATAC exc. ENCODE H3K27ac
% of fragments in peaks

Supplementary Figure 2. Quality control for all CUT&Tag, CUT&RUN, ENCODE ChIP samples. a) Fragment length distributions with and without duplicates. **b)** Percentage of fragments in sample peaks called with SEACR or MACS2, with or without duplicates, and narrow ENCODE H3K27ac and broad H3K27me3 ChIP-seq peaks. **c)** Percentage of CUT&Tag and ENCODE ChIP-seq reads in overlapping ENCODE and CUT&Tag peak regions. **d)** Percentage of sample reads in ENCODE H3K27ac ChIP-seq and ATAC-seq peaks. Figures have been expanded to include all analyzed samples and published datasets.

CnR: CUT&RUN; exc: excluding; HDup: high duplication rate sample; rmDup: duplicates removed.

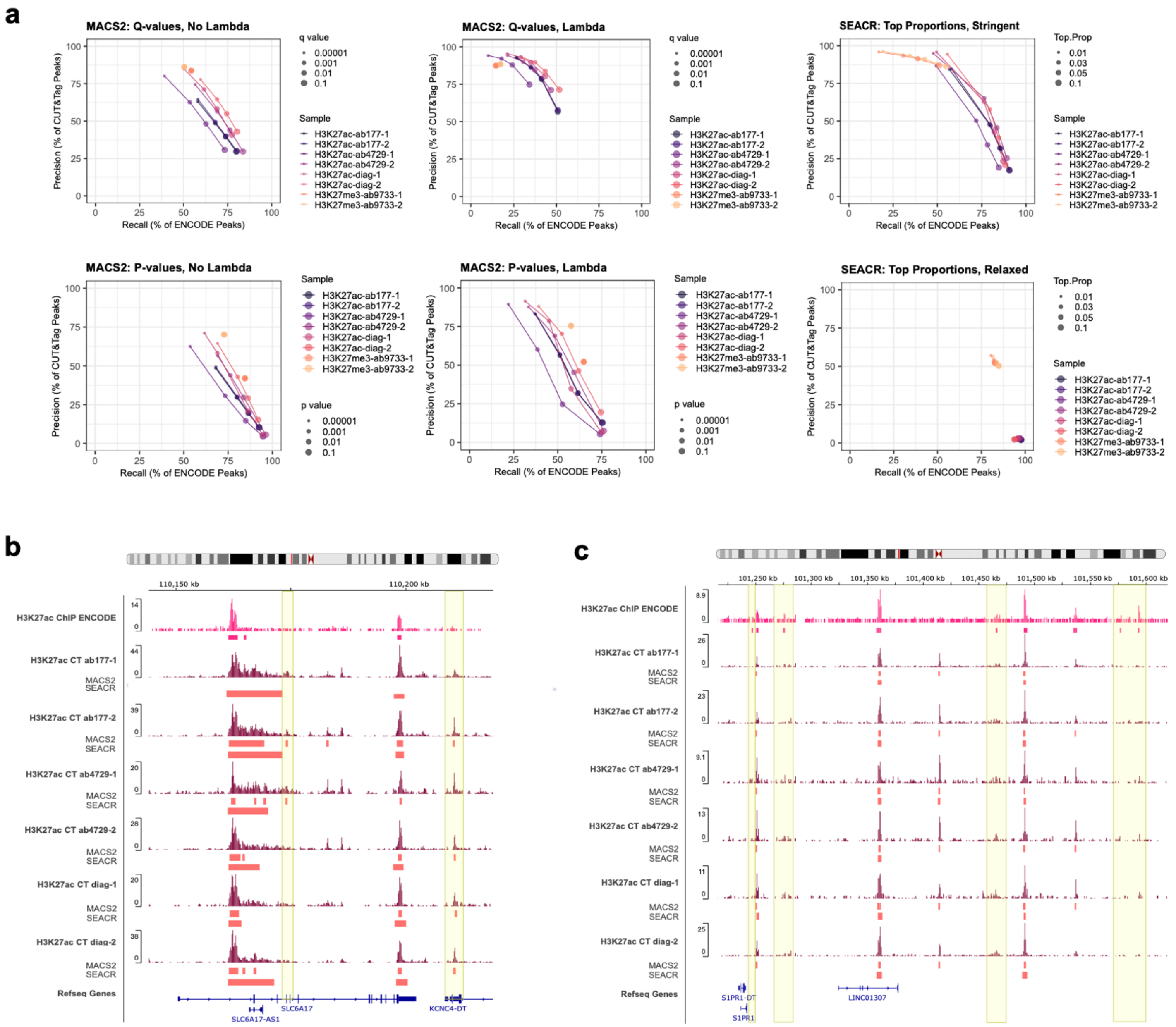
a
MACS2: Q-values, No Lambda
MACS2: Q-values, Lambda
SEACR: Top Proportions, Stringent
MACS2: P-values, No Lambda
MACS2: P-values, Lambda
SEACR: Top Proportions, Relaxed
Precision (% of CUT&Tag Peaks)
Recall (% of ENCODE Peaks)
q value
0.00001
0.001
0.01
0.1
p value
Top.Prop
0.03
0.05
Sample
H3K27ac-ab177-1
H3K27ac-ab177-2
H3K27ac-ab4729-1
H3K27ac-ab4729-2
H3K27ac-diag-1
H3K27ac-diag-2
H3K27me3-ab9733-1
H3K27me3-ab9733-2
b
c
110,150 kb
110,200 kb
101,250 kb
101,300 kb
101,350 kb
101,400 kb
101,450 kb
101,500 kb
101,550 kb
101,600 kb
H3K27ac ChIP ENCODE
H3K27ac CT ab177-1
H3K27ac CT ab177-2
H3K27ac CT ab4729-1
H3K27ac CT ab4729-2
H3K27ac CT diag-1
H3K27ac CT diag-2
MACS2
SEACR
Refseq Genes
SLC6A17-AS1
SLC6A17
KCNC4-DT
S1PR1-DT
S1PR1
LINC01307

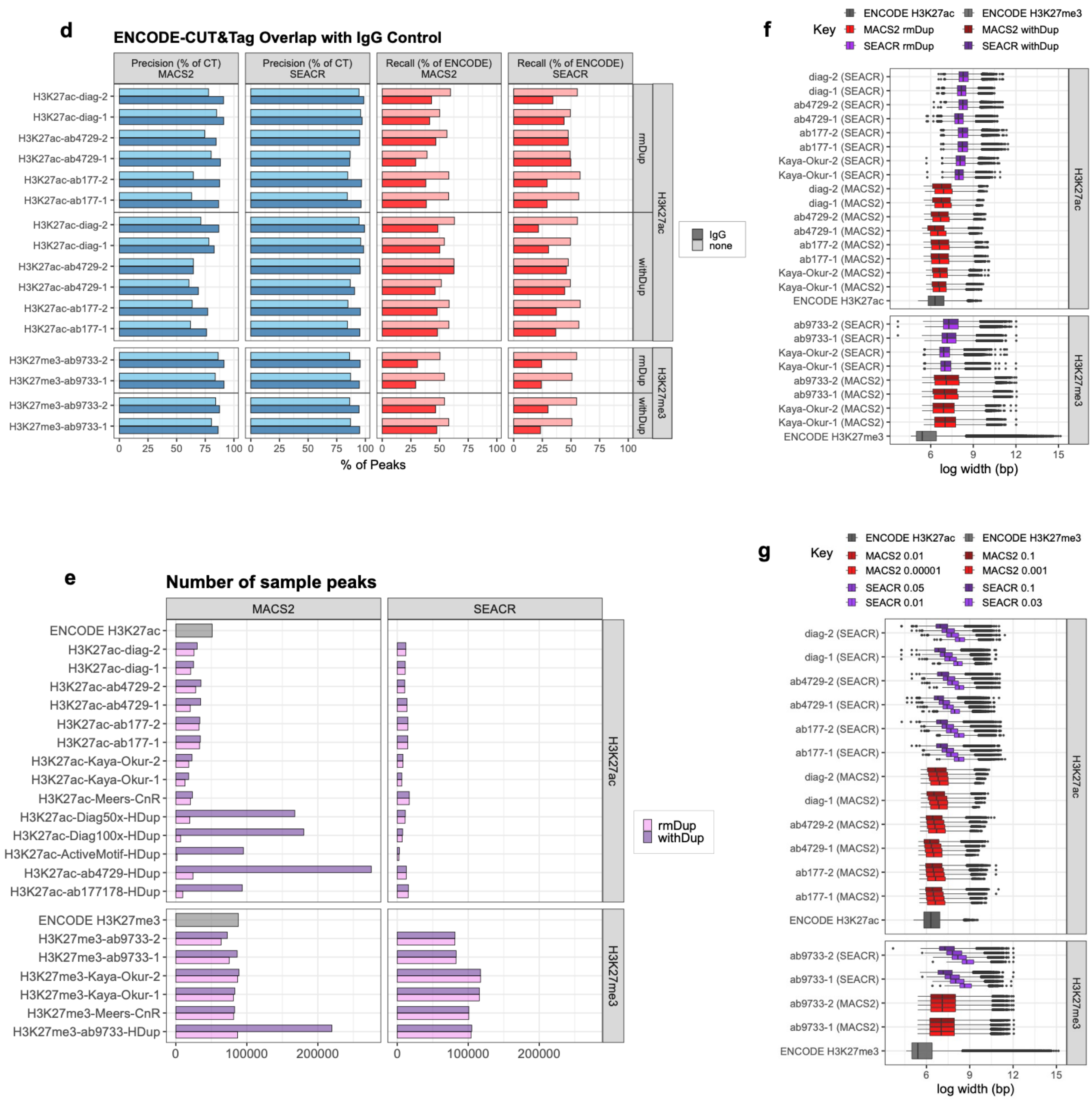

Supplementary Figure 3. Optimization of peak calling with MACS2 and SEACR. a) Precision and recall relative to ENCODE ChIP when varying q- and p-value thresholds, active and inactive local lambda for MACS2, and SEACR with stringent and relaxed settings. **b-c)** IGV tracks showing signal and peak regions defined by MACS2 and SEACR, where CUT&Tag peaks are missing in ENCODE (**b**) and ENCODE peaks missing in CUT&Tag (**c**). **d)** Precision and recall of ENCODE peaks with and without an IgG control. **e)** Number of peaks called with optimized peak calling parameters in

samples with and without high duplication rates. **f-g)** Boxplots showing the peak width distributions across samples and peak callers with and without duplicates (**f**) and across peak caller thresholds (**g**). The boxplots represent the median, first and third quartiles, whiskers correspond to 1.5 * the interquartile range (IQR), and dots to display outliers. Bp: base pairs; CT: CUT&Tag; IgG: Immunoglobulin G; kb: kilobases; rmDup: duplicates removed; Top.Prop: proportion of top peaks.

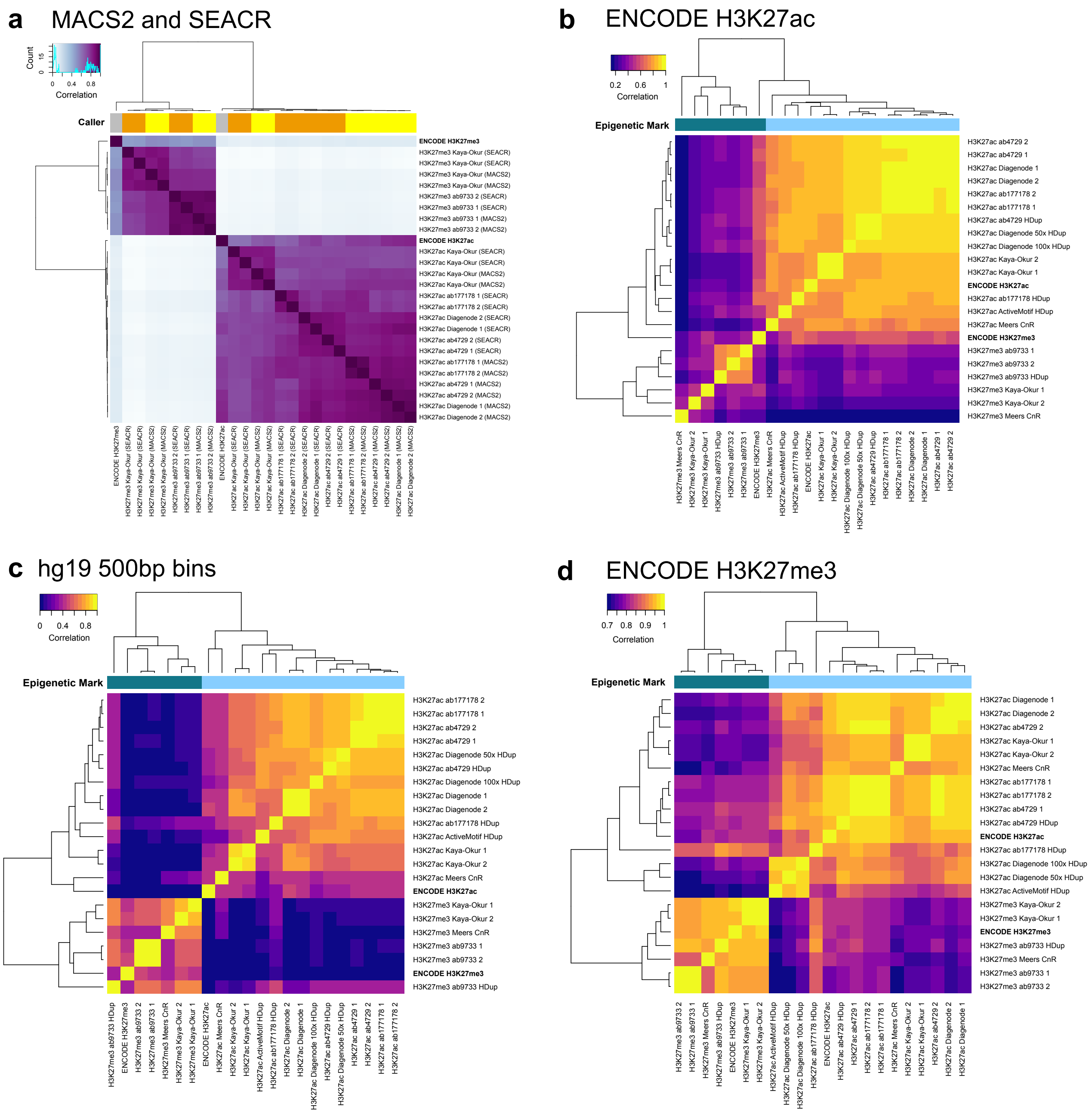
a MACS2 and SEACR
Count
Correlation
Caller
ENCODE H3K27me3
H3K27me3 Kaya-Okur (SEACR)
H3K27me3 Kaya-Okur (SEACR)
H3K27me3 Kaya-Okur (MACS2)
H3K27me3 Kaya-Okur (MACS2)
H3K27me3 ab9733 2 (SEACR)
H3K27me3 ab9733 1 (SEACR)
H3K27me3 ab9733 1 (MACS2)
H3K27me3 ab9733 2 (MACS2)
ENCODE H3K27ac
H3K27ac Kaya-Okur (SEACR)
H3K27ac Kaya-Okur (SEACR)
H3K27ac Kaya-Okur (MACS2)
H3K27ac Kaya-Okur (MACS2)
H3K27ac ab177178 1 (SEACR)
H3K27ac ab177178 2 (SEACR)
H3K27ac Diagenode 2 (SEACR)
H3K27ac Diagenode 1 (SEACR)
H3K27ac ab4729 2 (SEACR)
H3K27ac ab4729 1 (SEACR)
H3K27ac ab177178 1 (MACS2)
H3K27ac ab177178 2 (MACS2)
H3K27ac ab4729 1 (MACS2)
H3K27ac ab4729 2 (MACS2)
H3K27ac Diagenode 1 (MACS2)
H3K27ac Diagenode 2 (MACS2)
b ENCODE H3K27ac
0.2 0.6 1
Correlation
Epigenetic Mark
H3K27ac ab4729 2
H3K27ac ab4729 1
H3K27ac Diagenode 1
H3K27ac Diagenode 2
H3K27ac ab177178 2
H3K27ac ab177178 1
H3K27ac ab4729 HDup
H3K27ac Diagenode 50x HDup
H3K27ac Diagenode 100x HDup
H3K27ac Kaya-Okur 2
H3K27ac Kaya-Okur 1
ENCODE H3K27ac
H3K27ac ab177178 HDup
H3K27ac ActiveMotif HDup
H3K27ac Meers CnR
ENCODE H3K27me3
H3K27me3 ab9733 1
H3K27me3 ab9733 2
H3K27me3 ab9733 HDup
H3K27me3 Kaya-Okur 1
H3K27me3 Kaya-Okur 2
H3K27me3 Meers CnR
c hg19 500bp bins
0 0.4 0.8
Correlation
Epigenetic Mark
H3K27ac ab177178 2
H3K27ac ab177178 1
H3K27ac ab4729 2
H3K27ac ab4729 1
H3K27ac Diagenode 50x HDup
H3K27ac ab4729 HDup
H3K27ac Diagenode 100x HDup
H3K27ac Diagenode 1
H3K27ac Diagenode 2
H3K27ac ab177178 HDup
H3K27ac ActiveMotif HDup
H3K27ac Kaya-Okur 1
H3K27ac Kaya-Okur 2
H3K27ac Meers CnR
ENCODE H3K27ac
H3K27me3 Kaya-Okur 1
H3K27me3 Kaya-Okur 2
H3K27me3 Meers CnR
H3K27me3 ab9733 1
H3K27me3 ab9733 2
ENCODE H3K27me3
H3K27me3 ab9733 HDup
d ENCODE H3K27me3
0.7 0.8 0.9 1
Correlation
Epigenetic Mark
H3K27ac Diagenode 1
H3K27ac Diagenode 2
H3K27ac ab4729 2
H3K27ac Kaya-Okur 1
H3K27ac Kaya-Okur 2
H3K27ac Meers CnR
H3K27ac ab177178 1
H3K27ac ab177178 2
H3K27ac ab4729 1
H3K27ac ab4729 HDup
ENCODE H3K27ac
H3K27ac ab177178 HDup
H3K27ac Diagenode 100x HDup
H3K27ac Diagenode 50x HDup
H3K27ac ActiveMotif HDup
H3K27me3 Kaya-Okur 2
H3K27me3 Kaya-Okur 1
ENCODE H3K27me3
H3K27me3 ab9733 HDup
H3K27me3 Meers CnR
H3K27me3 ab9733 1
H3K27me3 ab9733 2

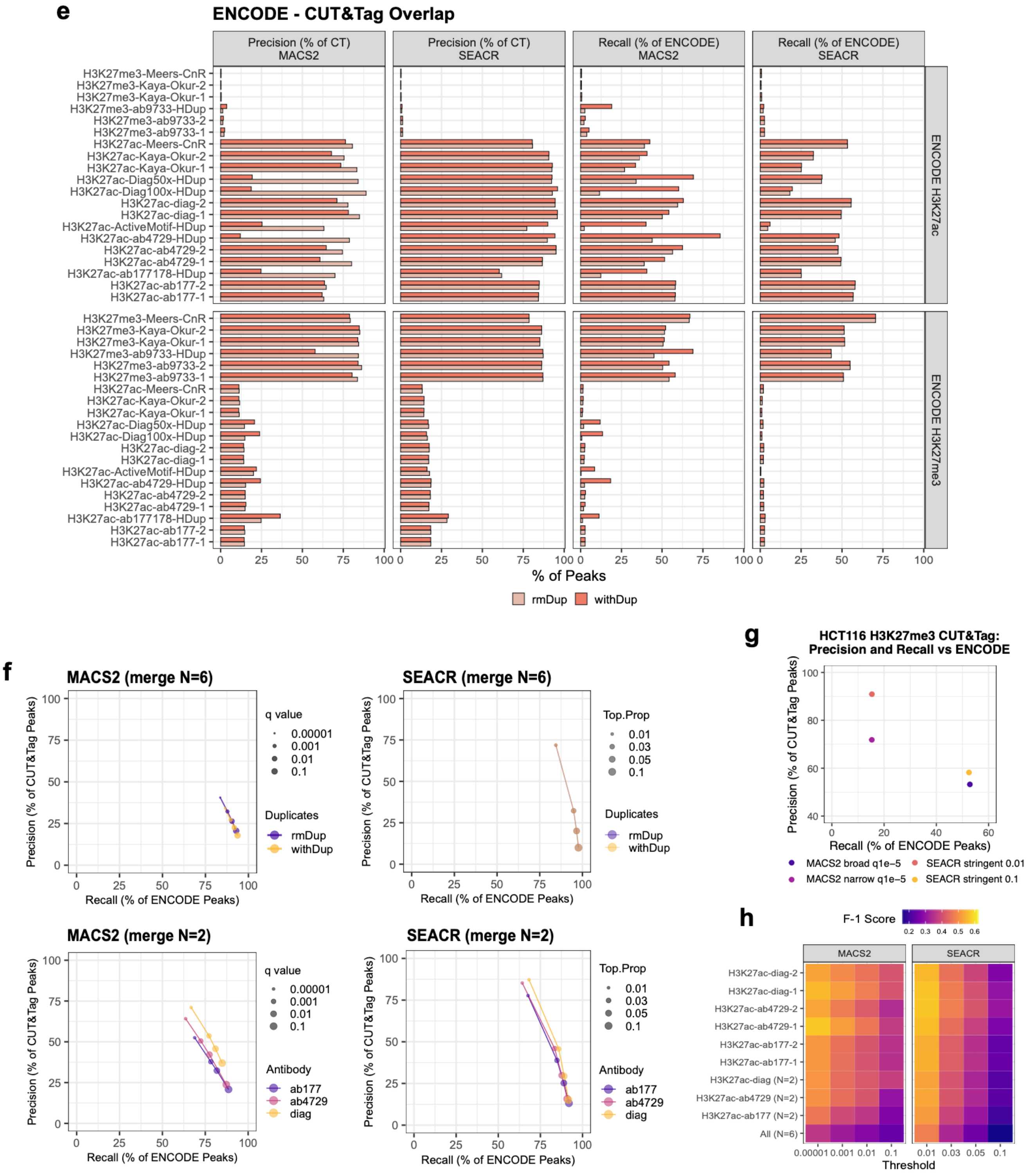

Supplementary Figure 4. Benchmarking CUT&Tag against ENCODE ChIP-seq. a) Peak correlations for H3K27ac and H3K27me3 CUT&Tag MACS2 (yellow) and SEACR

(orange) and ENCODE ChIP-seq (grey) genomic ranges. **b-d)** Read correlations across ENCODE H3K27ac ChIP-seq peak ranges, 500bp genome-wide bins and ENCODE H3K27me3 ChIP-seq peak ranges (H3K27ac samples: light blue; H3K27me3 samples: dark blue). **e)** Capture of ENCODE H3K27ac and H3K27me3 ChIP-seq peaks by CUT&Tag and CUT&RUN peaks called with SEACR or MACS2, with or without duplicates. **f)** Precision and recall of ENCODE capture with aggregate sample peak calling comprising of all internal CUT&Tag samples (top) or by antibody (bottom) at different MACS2 and SEACR thresholds. **g)** Precision and recall of ENCODE H3K27me3 peak capture in the HCT116 cell line. **h)** F-measures of precision and recall of tested H3K27ac antibodies at maximum read depth (‘full’), 8 million paired-end reads (‘8M’), and merged samples. Comparisons of peaks and reads are without duplicates unless stated otherwise.
Bp: base pairs; CnR: CUT&RUN; CT: CUT&Tag; HDup: high duplication rate sample; rmDup: duplicates removed.

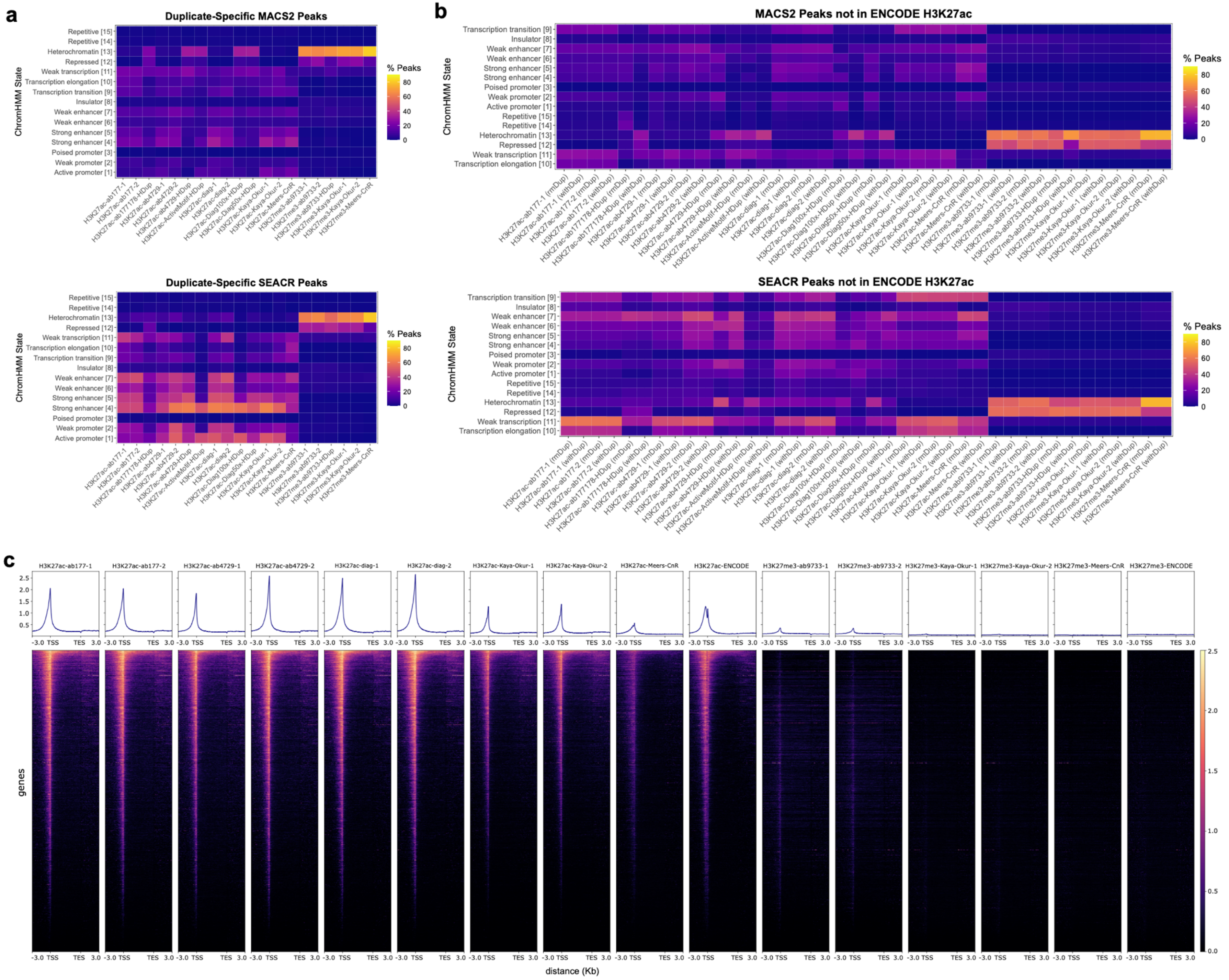

a
Duplicate-Specific MACS2 Peaks
ChromHMM State
Repetitive [15]
Repetitive [14]
Heterochromatin [13]
Repressed [12]
Weak transcription [11]
Transcription elongation [10]
Transcription transition [9]
Insulator [8]
Weak enhancer [7]
Weak enhancer [6]
Strong enhancer [5]
Strong enhancer [4]
Poised promoter [3]
Weak promoter [2]
Active promoter [1]
% Peaks
80
60
40
20
0
Duplicate-Specific SEACR Peaks
b
MACS2 Peaks not in ENCODE H3K27ac
SEACR Peaks not in ENCODE H3K27ac
c
H3K27ac-ab177-1
H3K27ac-ab177-2
H3K27ac-ab4729-1
H3K27ac-ab4729-2
H3K27ac-diag-1
H3K27ac-diag-2
H3K27ac-Kaya-Okur-1
H3K27ac-Kaya-Okur-2
H3K27ac-Meers-CnR
H3K27ac-ENCODE
H3K27me3-ab9733-1
H3K27me3-ab9733-2
H3K27me3-Kaya-Okur-1
H3K27me3-Kaya-Okur-2
H3K27me3-Meers-CnR
H3K27me3-ENCODE
-3.0 TSS TES 3.0
genes
distance (Kb)

Supplementary Figure 5. Functional assignments of CUT&Tag, CUT&RUN, and ENCODE signal. a-b) Chromatin state assignments of CUT&Tag peaks specific to duplicate-containing samples (**a**) and CUT&Tag peaks not in ENCODE H3K27ac (**b**). **c)** Heatmaps showing average read coverage around hg19 transcription start sites, with all samples subsampled to 2 million reads. Figures have been expanded to include all analyzed samples and published datasets.

CnR: CUT&RUN; HDup: high duplication rate sample; kb: kilobases; rmDup: duplicates removed, TES: transcription end site; TSS: transcription start site.

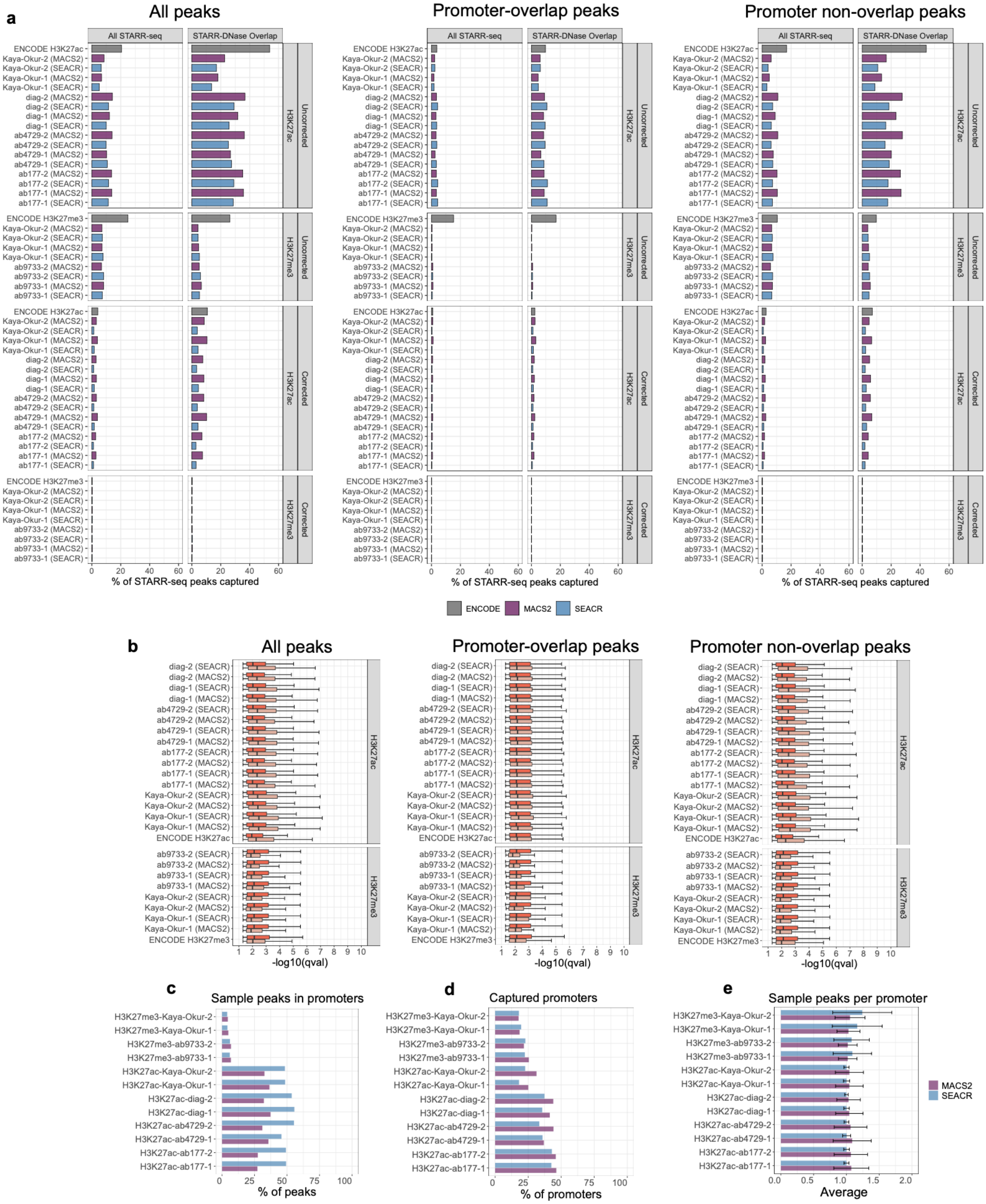
a
All peaks
Promoter-overlap peaks
Promoter non-overlap peaks
All STARR-seq
STARR-DNase Overlap
Uncorrected
Corrected
H3K27ac
H3K27me3
ENCODE H3K27ac
Kaya-Okur-2 (MACS2)
Kaya-Okur-2 (SEACR)
Kaya-Okur-1 (MACS2)
Kaya-Okur-1 (SEACR)
diag-2 (MACS2)
diag-2 (SEACR)
diag-1 (MACS2)
diag-1 (SEACR)
ab4729-2 (MACS2)
ab4729-2 (SEACR)
ab4729-1 (MACS2)
ab4729-1 (SEACR)
ab177-2 (MACS2)
ab177-2 (SEACR)
ab177-1 (MACS2)
ab177-1 (SEACR)
ENCODE H3K27me3
ab9733-2 (MACS2)
ab9733-2 (SEACR)
ab9733-1 (MACS2)
ab9733-1 (SEACR)
0 20 40 60
% of STARR-seq peaks captured
ENCODE
MACS2
SEACR
b
All peaks
Promoter-overlap peaks
Promoter non-overlap peaks
1 2 3 4 5 6 7 8 9 10
-log10(qval)
c
Sample peaks in promoters
H3K27me3-Kaya-Okur-2
H3K27me3-Kaya-Okur-1
H3K27me3-ab9733-2
H3K27me3-ab9733-1
H3K27ac-Kaya-Okur-2
H3K27ac-Kaya-Okur-1
H3K27ac-diag-2
H3K27ac-diag-1
H3K27ac-ab4729-2
H3K27ac-ab4729-1
H3K27ac-ab177-2
H3K27ac-ab177-1
0 25 50 75 100
% of peaks
d
Captured promoters
% of promoters
e
Sample peaks per promoter
0.0 0.5 1.0 1.5 2.0
Average
MACS2
SEACR

Supplementary Figure 6. Capture of promoters and STARR-seq enhancers by CUT&Tag. a) Genome-wide overlap percentage of total STARR-seq peaks with CUT&Tag (deduplicated) sample peaks and ENCODE ChIP-seq peaks (left), both without (top) and with (bottom) correction of total genomic coverage, and STARR-seq peaks restricted to those overlapping ENCODE K562 DNase-seq peaks (right). **b**) Significance −log10(q) values of DNase-overlapping STARR-seq peaks captured and not captured by CUT&Tag and ChIP-seq. Boxplot represents the median, first and third quartiles, whiskers correspond to 1.5 * the interquartile range (IQR). Welch two-sided t-test used to calculate the significance of the difference between captured and missed vvvalueluelues in each peak set. **c**) Percentage of CUT&Tag peaks overlapping promoters. **d**) Percentage of total reference promoters captured by CUT&Tag peaks. **e**) Average number of CUT&Tag sample peaks overlapping a captured promoter. Error bars represent mean±SD across CUT&Tag peaks for a given antibody and peak caller.
