# Supplementary material for: CUT&Tag recovers up to half of ENCODE ChIP-seq histone acetylation peaks
Source: Nat Commun. 2025 Mar 27;16:2993. doi: 10.1038/s41467-025-58137-2 (PMC11950320; doi:10.1038/s41467-025-58137-2)
Supplement: Supplementary file 2 — Reporting Summary [file 41467_2025_58137_MOESM2_ESM.pdf]

nature portfolio

Corresponding author(s): Sarah J. Marzi

Last updated by author(s): Mar 12, 2025

Reporting Summary

Nature Portfolio wishes to improve the reproducibility of the work that we publish. This form provides structure for consistency and transparency in reporting. For further information on Nature Portfolio policies, see our Editorial Policies and the Editorial Policy Checklist.

Statistics

For all statistical analyses, confirm that the following items are present in the figure legend, table legend, main text, or Methods section.

n/a	Confirmed	
☐	☒	The exact sample size (*n*) for each experimental group/condition, given as a discrete number and unit of measurement
☐	☒	A statement on whether measurements were taken from distinct samples or whether the same sample was measured repeatedly
☐	☒	The statistical test(s) used AND whether they are one- or two-sided<br>*Only common tests should be described solely by name; describe more complex techniques in the Methods section.*
☒	☐	A description of all covariates tested
☐	☒	A description of any assumptions or corrections, such as tests of normality and adjustment for multiple comparisons
☐	☒	A full description of the statistical parameters including central tendency (e.g. means) or other basic estimates (e.g. regression coefficient) AND variation (e.g. standard deviation) or associated estimates of uncertainty (e.g. confidence intervals)
☐	☒	For null hypothesis testing, the test statistic (e.g. *F*, *t*, *r*) with confidence intervals, effect sizes, degrees of freedom and *P* value noted<br>*Give P values as exact values whenever suitable.*
☒	☐	For Bayesian analysis, information on the choice of priors and Markov chain Monte Carlo settings
☒	☐	For hierarchical and complex designs, identification of the appropriate level for tests and full reporting of outcomes
☐	☒	Estimates of effect sizes (e.g. Cohen's *d*, Pearson's *r*), indicating how they were calculated

Our web collection on statistics for biologists contains articles on many of the points above.

Software and code

Policy information about availability of computer code

Data collection	CUT&Tag nuclei processing:<br>Bench top CUT&Tag was performed as previously described (https://www.protocols.io/view/bench-top-cut-amp-tag-bcuhiwt6). Exponentially growing K562 cells were harvested, counted and centrifuged for 3 min at 600g at room temperature (RT). 500,000 cells per condition were washed twice in 1 mL Wash Buffer (20 mM HEPES-KOH pH 7.5, 150 mM NaCl, 0.5 mM Spermidine, 1x Protease inhibitor cocktail; Roche 11836170001). Nuclei were extracted by incubating cells for 10 minutes on ice in 200 µL/sample of cold Nuclei Extraction buffer (NE buffer: 20 mM HEPES-KOH pH 7.9, 10 mM KCl, 0.1% Triton X-100, 20% Glycerol, 0.5 mM Spermidine, 1x Protease Inhibitor cocktail). Following incubation in NE buffer, nuclei were centrifuged for 3 min at 600g at RT, then resuspended in 100 µL cold NE buffer. Concanavalin A coated magnetic beads (Bangs Laboratories BP531) were prepared as previously described and 11 µL of activated beads were added per sample into PCR strip tubes and incubated at RT with gentle rocking for 10 min. Beads were placed on a magnetic rack and unbound supernatant was discarded. Bead bound nuclei were resuspended in 50 µL Dig-wash Buffer (20 mM HEPES pH 7.5, 150 mM NaCl, 0.5 mM Spermidine, 1× Protease inhibitor cocktail, 0.05% Digitonin) with 2 mM EDTA and 0.1% BSA. 1:50/1:100/1:200 dilution of primary antibody was added, followed by a gentle vortex and brief spin. Primary antibody incubation was conducted on a rotating platform overnight at 4°C. Primary antibody solution was removed by placing the PCR tube on a magnetic rack, allowing the solution to fully clear, then removing the supernatant. Next, the appropriate secondary antibody, Guinea Pig anti-Rabbit IgG antibody for a rabbit primary antibody, was added at 1:100 in Dig-Wash buffer and incubated at RT with rotation for 30-60 min. Nuclei were washed twice in 200 µL Dig-Wash buffer using a magnetic rack to remove unbound antibodies in supernatant. Nuclei were resuspended in 50 µL Dig-med Buffer (20 mM HEPES pH 7.5, 300 mM NaCl, 0.5 mM Spermidine, 1× Protease inhibitor cocktail, 0.05% Digitonin), then 1:20 CUTANA™ pAG-Tn5 (Epicypher 15-1017), or 1:250 pA-Tn5 Transposase - loaded (Diagenode C01070001) was added, gently mixed and spun down. pA-Tn5 binding occurred at RT for 1 hour on a rotating platform. To remove unbound pA-Tn5, nuclei were washed twice in 200 µL Dig-med Buffer. Nuclei were then resuspended in 50 µL Tagmentation buffer (10 mM $MgCl_2$ in Dig-med Buffer) and incubated at 37°C for 1 hour to activate transposase enzymatic activity. Next, either column or sodium dodecyl sulfate (SDS) based DNA extraction was conducted.

Column DNA extraction:
To stop tagmentation and solubilise DNA fragments, the following were added to each 50 µL sample: 1.68 µL 0.5M ethylenediaminetetraacetic acid (EDTA), 0.5 µL 10% SDS, 0.44 µL 10 mg/mL Proteinase K. The samples were briefly mixed and vortexed at full speed for ~2 seconds, then incubated at 55°C for 1 hour to digest the DNA. After a quick spin, tubes were placed on a magnetic rack and solution was allowed to clear. Supernatant was carefully transferred to a new 1.5 mL microcentrifuge tube, then sample processing protocol of ChIP DNA Clean & Concentrator (Zymo Research D5205) was executed, eluting with 21 µL Elution Buffer.

SDS-based DNA extraction:
Following tagmentation at 37°C for 1 hour, PCR tubes were placed on a magnetic rack and solution was allowed to clear. Supernatant was removed carefully, then beads were resuspended thoroughly in 50 µL [tris(hydroxymethyl)methylamino]propanesulfonic acid (TAPS) Buffer (10 mM TAPS pH 8.5, 0.2 mM EDTA) at RT. Tubes were returned to a magnetic rack and supernatant was removed. 5 µL SDS Release Buffer (10 mM TAPS pH 8.5, 0.1% SDS) was added at RT to each sample and tubes were vortexed at full speed for ~10 seconds. After a quick spin, ensuring no beads are stuck to the side of the tubes, samples were incubated at 58°C for 1 hour. Next, 15 µL SDS Quench Buffer (0.67% Triton-X 100 in Molecular grade H2O) was added at RT and vortexed at maximum speed to neutralise the SDS prior to PCR library amplification.

CUT&Tag PCR-based library amplification:
For library amplification in PCR tube format, 21 µL DNA was combined with 2 µL of universal i5 and uniquely barcoded i7 primer50 where a different barcode was used for each sample that was intended to be pooled together. 25 µL NEBNext HiFi 2× PCR Master mix was added, then the sample was gently mixed through and spun down. The sample was placed in a Thermocycler with heated lid following these conditions: 72°C for 5 min (gap filling); 98°C for 30 s; 11-15 cycles of 98°C for 10 s and 63°C for 30 s; final extension at 72°C for 1 min; and hold at 4°C. Following PCR, bead cleanup was conducted by addition of 1.1x Ampure XP beads (Beckman Coulter). Library and beads were mixed thoroughly, then spun down and incubated at RT for 10-15 min. Beads were gently washed twice with freshly prepared 80% ethanol using a magnetic rack, then the library was eluted with 20-30 µL 10 mM Tris-HCl pH 8.0 at RT.

Sequencing:
Final library size distributions were assessed by Agilent 2100 Bioanalyser and Agilent 4200 TapeStation for quality control before sequencing. Libraries were pooled to achieve equal representation of the desired final library size range (equimolar pooling based on Bioanalyser/ TapeSation signal in the 150bp to 800bp range). Paired-end Illumina sequencing using the HiSeq 4000 PE75 strategy was conducted on barcoded libraries at Imperial Biomedical Research Centre (BRC) Genomics Facility following manufacturer's protocols.

Data analysis

Code used for data processing and analysis has been deposited in a dedicated Github repository: https://github.com/Marzi-lab/CUTnTag-benchmarking-analysis

Briefly, sample fastqs were subjected to adapter trimming, alignment, removal/retention of duplicate reads, and peak calling with different peak callers. Command line tools were also used at this stage to perform analyses including read-level correlation and estimation of read distribution and enrichment. The following packages were used for data processing:
Cutadapt (version 1.18)
TrimGalore (version 0.6.6)
Bowtie (version 2.2.9)
Picard (version 2.6.0)
bedtools (version 2.25.0)
deeptools (version 3.5.4)
samtools (version 1.3.1)
SEACR (version 1.3)
MACS2 (version 2.2.9.1)
HOMER (version 4.11.1)

Downstream analysis comprised quality control, appraisal of different peak calling methods, benchmarking of different CUT&Tag antibodies and of CUT&Tag against ENCODE ChIP-seq, and functional characterisation of CUT&Tag peaks. The following R packages were used for downstream analysis:
GenomicRanges (version 1.50.2)
IRanges (version 2.32.0)
genomation (version 1.30.0)
clusterProfiler (version 4.6.2)
rtracklayer (version 1.58.0)
chromVAR (version 1.20.2)
ChIPseeker (version 1.34.1)
DiffBind (version 3.8.4)
BRGenomics (version 1.10.0)
marge (version 0.0.4.9999)
TxDb.Hsapiens.UCSC.hg19.knownGene database (version 3.2.2)

For manuscripts utilizing custom algorithms or software that are central to the research but not yet described in published literature, software must be made available to editors and reviewers. We strongly encourage code deposition in a community repository (e.g. GitHub). See the Nature Portfolio guidelines for submitting code & software for further information.

Data

Policy information about availability of data

All manuscripts must include a data availability statement. This statement should provide the following information, where applicable:

- Accession codes, unique identifiers, or web links for publicly available datasets
- A description of any restrictions on data availability
- For clinical datasets or third party data, please ensure that the statement adheres to our policy

The CUT&Tag data generated in this study have been deposited in the Gene Expression Omnibus (GEO) database under accession code GSE286492 [https://www.ncbi.xyz/geo/query/acc.cgi?acc=GSE286492]. The bigwig, bedgraph and peak data generated in this study can be found at: https://data.cyverse.org/dav-anon/iplant/home/paulinaurbana/H3K27_CUT%26Tag_Benchmark/. The UCSC bigwig tracks data generated in this study can be found at: https://genome.ucsc.edu/s/pu1918/CUT_and_Tag_benchmarking.

Human research participants

Policy information about studies involving human research participants and Sex and Gender in Research.

Reporting on sex and gender	*Use the terms sex (biological attribute) and gender (shaped by social and cultural circumstances) carefully in order to avoid confusing both terms. Indicate if findings apply to only one sex or gender; describe whether sex and gender were considered in study design whether sex and/or gender was determined based on self-reporting or assigned and methods used. Provide in the source data disaggregated sex and gender data where this information has been collected, and consent has been obtained for sharing of individual-level data; provide overall numbers in this Reporting Summary. Please state if this information has not been collected. Report sex- and gender-based analyses where performed, justify reasons for lack of sex- and gender-based analysis.*
Population characteristics	*Describe the covariate-relevant population characteristics of the human research participants (e.g. age, genotypic information, past and current diagnosis and treatment categories). If you filled out the behavioural & social sciences study design questions and have nothing to add here, write "See above."*
Recruitment	*Describe how participants were recruited. Outline any potential self-selection bias or other biases that may be present and how these are likely to impact results.*
Ethics oversight	*Identify the organization(s) that approved the study protocol.*

Note that full information on the approval of the study protocol must also be provided in the manuscript.

Field-specific reporting

Please select the one below that is the best fit for your research. If you are not sure, read the appropriate sections before making your selection.

[x] Life sciences [ ] Behavioural & social sciences [ ] Ecological, evolutionary & environmental sciences

For a reference copy of the document with all sections, see nature.com/documents/nr-reporting-summary-flat.pdf

Life sciences study design

All studies must disclose on these points even when the disclosure is negative.

Sample size	3 technical replicates and 3 biological replicates were used for each condition for all qPCR analyses. 2 biological replicates were used for CUT&Tag sequencing of the main 3 H3K27ac antibodies and H3K27me3.
Data exclusions	N/A
Replication	3 technical replicates and 3 biological replicates were used for each condition for all qPCR analyses. 2 biological replicates were used for CUT&Tag sequencing of the main 3 H3K27ac antibodies and H3K27me3.
Randomization	Samples treated with a particular condition were known and not randomised. Samples were treated uniformly using identical experimental and computational pipeline per analysis.
Blinding	Samples treated with a particular condition were known and not blinded to the researcher. Samples were treated uniformly using identical experimental and computational pipeline per analysis.

Reporting for specific materials, systems and methods

We require information from authors about some types of materials, experimental systems and methods used in many studies. Here, indicate whether each material, system or method listed is relevant to your study. If you are not sure if a list item applies to your research, read the appropriate section before selecting a response.

Materials & experimental systems

n/a	Involved in the study
☐	☒ Antibodies
☐	☒ Eukaryotic cell lines
☒	☐ Palaeontology and archaeology
☒	☐ Animals and other organisms
☒	☐ Clinical data
☒	☐ Dual use research of concern

Methods

n/a	Involved in the study
☒	☐ ChIP-seq
☒	☐ Flow cytometry
☒	☐ MRI-based neuroimaging

Antibodies

Antibodies used	The following antibodies were used: Guinea Pig anti-Rabbit IgG (Heavy & Light Chain) Preabsorbed antibody (Antibodies-Online ABIN101961), H3K27me3 (Cell Signaling Technology, 9733, Lot 14), H3K27ac (Abcam ab177178, Lot GR3202987-5), H3K27ac (Active Motif 39133, Lot 16119013), H3K27ac (Abcam ab4729, Lot G3374555-1), H3K72ac (Diagenode C15410196, Lot A1723-0041D).
Validation	Guinea Pig anti-Rabbit IgG (Heavy & Light Chain) Preabsorbed antibody (Antibodies-Online ABIN101961), H3K27me3 (Cell Signaling Technology, 9733, Lot 14) - used and validated for CUT&Tag in Kaya-Okur, H. S. et al. CUT&Tag for efficient epigenomic profiling of small samples and single cells. Nat. Commun. 10, 1930 (2019); Kaya-Okur, H. S., Janssens, D. H., Henikoff, J. G., Ahmad, K. & Henikoff, S. Efficient low-cost chromatin profiling with CUT&Tag. Nat. Protoc. 15, 3264–3283 (2020).<br><br>H3K27ac (Abcam ab177178, Lot GR3202987-5) - used and validated for CUT&Tag in Bartosovic, M., Kabbe, M. & Castelo-Branco, G. Single-cell CUT&Tag profiles histone modifications and transcription factors in complex tissues. Nat. Biotechnol. 39, 825–835 (2021).<br><br>H3K27ac (Abcam ab4729, Lot G3374555-1) - used and validated for CUT&Tag in Zhu, C. et al. Joint profiling of histone modifications and transcriptome in single cells from mouse brain. Nat. Methods 18, 283–292 (2021).<br><br>H3K27ac (Active Motif 39133, Lot 16119013) - used and validated for CUT&Tag by Active Motif https://www.activemotif.com/catalog/details/39133/histone-h3-acetyl-lys27-antibody-pab<br><br>H3K72ac (Diagenode C15410196, Lot A1723-0041D) - use and validated for CUT&Tag by Diagenode https://www.diagenode.com/en/p/h3k27ac-polyclonal-antibody-premium-50-mg-18-ml

Eukaryotic cell lines

Policy information about cell lines and Sex and Gender in Research

Cell line source(s)	Human K562 cells were obtained from ATCC (Manassas, VA, Catalog #CCL-243)
Authentication	K562 cells purchased from manufacturer were not authenticated.
Mycoplasma contamination	Mycoplasma was tested to be negative for all cellular input reported using Mycoplasma Detection Kit (Jena Bioscience PP-401) following manufacturer's instructions.
Commonly misidentified lines (See ICLAC register)	N/A
